# Analysing the Effects of Different Types of Exercise on Dyspnoea and Fatigue in Adults through COPD-Systematic Review and Meta-Analysis of Randomised Clinical Trials

**DOI:** 10.3390/healthcare11101449

**Published:** 2023-05-16

**Authors:** Nuno Couto, Luís Cid, Susana Alves, João Paulo Brito, Nuno Pimenta, Teresa Bento

**Affiliations:** 1 Sport Sciences School of Rio Maior, Polytechnic of Santarém (ESDRM-IPSantarém), 2040-413 Rio Maior, Portugal; luiscid@esdrm.ipsantarem.pt (L.C.); salves@esdrm.ipsantarem.pt (S.A.); jbrito@esdrm.ipsantarem.pt (J.P.B.); npimenta@esdrm.ipsantarem.pt (N.P.); teresabento@esdrm.ipsantarem.pt (T.B.); 2 Research Center in Sports Sciences, Health Sciences and Human Development (CIDESD), 5000-556 Vila Real, Portugal; 3 Life Quality Research Center (CIEQV), 2040-413 Santarém, Portugal; 4 Interdisciplinary Centre for the Study of Human Performance (CIPER), Faculty of Human Kinetics, 1495-207 Lisbon, Portugal; 5 The Interdisciplinary Health Research Centre, Catholic University of Portugal, 1649-023 Lisbon, Portugal

**Keywords:** COPD, pulmonary rehabilitation, aerobic exercise, resistance exercise, combined exercise, randomised controlled trials, systematic review, meta-analysis

## Abstract

Background: Chronic obstructive pulmonary disease (COPD) is a heterogeneous lung condition, the main symptoms of which are dyspnoea and fatigue. Though exercise has been recommended for subjects with COPD, its benefits remain unclear. The aim of this study was to summarise, through a systematic review and meta-analysis, the available evidence on the effects of aerobic, resistance, stretching, and combined exercise on the main symptoms of COPD. Methods: Search was performed using the electronic databases PubMed and Web of Science. Randomised controlled trials (RCTs) with interventions based on aerobic, resistance and/or combined exercise published until July 2022 were identified. The effects were summarised based on standardised mean differences (95% confidence intervals) using random and fixed effect models. Results: Eight studies were selected, including a total of 375 subjects. The results obtained showed that resistance exercise, aerobic exercise and combined exercise seem to improve dyspnoea and fatigue symptoms in COPD subjects. Conclusions: In general, we can conclude that exercise-based interventions appear to improve the main COPD symptoms and may benefit quality of life in this population.

## 1. Introduction

Chronic obstructive pulmonary disease (COPD) is a heterogeneous lung condition characterised by chronic respiratory symptoms (dyspnoea, cough, sputum production, exacerbations, etc.) caused by abnormalities of the airways (bronchitis, and bronchiolitis) and/or alveoli (emphysema) that result in persistent and often progressive, airflow obstruction, categorised into four levels of severity: level one (mild); level two (moderate); level three (severe); and level four (very severe) [1]. It was reported that COPD was the third leading cause of death in 2019, affecting both men and women worldwide, and may foster a considerable financial burden due to the limitation of workplace and home productivity and the costs of medical treatment [2]. There is no known cure for COPD, and as it progresses, people find it more difficult to carry out their normal daily activities, often due to breathlessness [2]. Fatigue and dyspnoea are the most common symptoms of COPD [1,3].

There are several actions that people with COPD can take to improve their overall health and that can help control the disease, such as quitting smoking, getting vaccinated against pneumonia, influenza and coronavirus, and doing regular exercise [2]. Exercise appears as a fundamental component of effective pulmonary rehabilitation programmes in all stages of COPD and can minimise exertional dyspnoea through the enhancement of ventilatory efficiency, reduction in fatigue, and improvement of cardiovascular and peripheral muscle function, exercise tolerance, health, and quality of life [4,5,6,7,8]. Exercise and pulmonary rehabilitation represent a real value for money as an intervention in moderate and severe COPD. The importance of this is particularly evident at a time when global financial austerity is affecting healthcare services [9].

Exercise participation is recommended in subjects suffering from COPD, and the prescription should consider disease severity, control of the condition, and other related or unrelated comorbidities [5]. Exercise recommendations in COPD patients are contentious and ambiguous. The British Thoracic Society [10] recommended aerobic exercise combined with resistance exercise, twice a week, in 60 min sessions with an intensity range of 50–85%. Aerobic exercise (continuous or intermittent) at 60–80% of the symptom-limited maximum work or heart rate is preferred [11], or a Borg-rated dyspnoea or fatigue score of 4 to 6 (moderate to severe) is favoured, according to Cooke et al. [12]. According to ACSM [13] and ATS/ERS [14], resistance and flexibility training should be encouraged in individuals with COPD and should follow the same rationale as suggested for healthy adults and/or older adults.

Previous systematic reviews have studied the effects of exercise on COPD symptoms [15,16]. A systematic review by Paixão et al. [15] observed that unsupervised exercise interventions improve dyspnoea and exercise capacity, however, they also identified the small number of studies, the large diversity of designs, outcomes and outcomes measures, and the high heterogeneity as limitations of their study. On the other hand, Paneroni et al. [16], who analysed the effects of the different types of exercise on fatigue, concluded that their study provided low-quality evidence of a positive impact of different exercise programmes on perceived fatigue in patients with COPD. Salcedo et al. [8] observed that whole-body exercise is effective for improving pulmonary function in adults with chronic disease. Cheng et al. [17], in a cohort study, found that the protective effects of exercise in subjects with COPD appear at considerably lower levels than the general physical activity recommendations. Considering the above-mentioned studies, further research is necessary to determine the optimal exercise training characteristics to maximise functional improvement, as well as a systematic review of the literature regarding the effectiveness of exercise training on dyspnoea and fatigue in patients with COPD. Accordingly, we conducted a systematic review with meta-analyses, aiming to study the effect of exercise on the main symptoms of COPD, dyspnoea and fatigue, and to contribute to the clarification of the type of exercise that could have a more positive impact on these patients.

## 2. Materials and Methods

A systematic review and meta-analysis were conducted, using PubMed and Web of Science (WOS) electronic databases, to identify longitudinal studies published until 31 July 2022, following the PRISMA protocol [18]. From an initial exploratory search, the descriptors were defined to identify the studies. The search was performed for the period between June and July 2022, using the U.S. National Library of Medicine’s Medical Subject Headings terms related to Exercise (#1) and COPD (#2) (#1 Additionally, #2) in PubMed (1300 references) and in the WOS (1924 references) Databases in all fields, in English, with no restriction to the date of publication. Potentially relevant articles were searched in the reference lists of the manuscripts obtained in the search, and other systematic reviews and meta-analyses were included if they contained relevant data. The present study was registered in the PROSPERO database, under the number CRD42022355447.

### 2.1. Eligibility Criteria

The eligibility criteria of the studies were determined according to the PICOS (Population, Intervention, Comparison, Outcomes, and Study Design) [19] strategy, as shown in Table 1.

Studies were excluded if (1) they included participants with age < 18 years old; (2) participants performed an intervention other than exercise; (3) they did not compare the results of EG with CG; (4) they did not clearly describe the exercise programme characteristics; (5) they included participants with any associated disease (i.e., cancer, dementia, diabetes, etc.) or non-physically independent; (6) they were written in a language other than English, Portuguese, or Spanish; and (7) the articles were not original (e.g., reviews, letters to editors, trial registrations, proposals for protocols, editorials, book chapters, and conference abstracts).

### 2.2. Study Identification

After removing all duplicates, studies were independently screened by two researchers, based on titles and abstracts, followed by a selection through reading of the full text of the manuscripts. In case of any conflict regarding the inclusion or exclusion of RCTs, consensus was achieved by consulting a third reviewer.

### 2.3. Data Extraction

The following data were extracted from the selected studies: country of origin, authors, design, number of participants, age, gender, type of exercise, intensity, symptoms, outcomes and conclusions of the study (Table 2).

### 2.4. Quality of Study and Risk of Bias

The quality of the included studies and the issues related to the risk of bias were evaluated using the Cochrane Collaboration Risk of Bias Tool [28]. Two reviewers assessed the quality of the studies, and differences between both reviewers were resolved by mutual agreement or by consulting a third reviewer. 

### 2.5. GRADE Assessment

The strength of the evidence was assessed using the Grading of Recommendations, Assessment, Development and Evaluation (GRADE) [29] system through GRADEpro. Quality of evidence for meta-analyses began at the high level and was downgraded to lower levels of evidence when risk of bias, inconsistency, indirectness, imprecision, or publication bias were presented. Two investigators rated the certainty of each treatment comparison independently and resolved discrepancies via discussions and, if necessary, consulted with a third party.

### 2.6. Data Synthesis and Analysis

Meta-analyses were performed for studies that compared exercise interventions using the Cochrane Review Manager Software (RevMan 5.4.1). The standard mean difference of dyspnoea and fatigue measurements pre and post intervention were calculated. The standard deviation (SD) of the mean difference, when not presented in the studies, was estimated using procedures recommended by the Cochrane handbook [28]. When there was significant heterogeneity (*p* ≤ 0.05), the randomised effect was used. When there was no significant heterogeneity (*p* > 0.05), we used fixed effects. The effect sizes were interpreted as very small (≥0.01), small (≥0.20), medium (≥0.50), large (≥0.80), very large (≥1.20) and huge (≥2.0) [30].

Studies’ heterogeneity was assessed by calculating the following statistics: (i) Tau^2^, (ii) Chi^2^, and (iii) *I*^2^. The following classification was used to evaluate the *I*^2^ (i.e., described inconsistency between trials): lower than 50% represents low heterogeneity; 50–74% represents substantial heterogeneity; and 75% and higher represents considerable heterogeneity [31].

## 3. Results

### 3.1. Results of the Systematic Literature Search Are Summarised in the PRISMA Flowchart (Figure 1)

Out of 3224 initially screened records, 843 were duplicates, and 2150 were excluded after title and abstract review. A full-text review of 231 studies allowed the identifications of eight studies [20,21,22,23,24,25,26,27] which met the inclusion and exclusion criteria and were included for review and meta-analysis.

**Figure 1:**
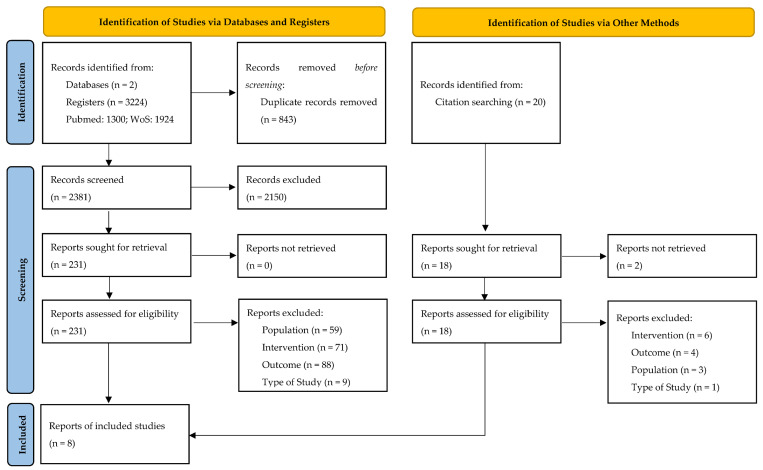
PRISMA flow diagram.

### 3.2. Study Characteristics

The total sample at baseline consisted of 375 female and male subjects over 18 years old, with moderate-to-severe COPD disease, at stable status, and an average forced expiratory volume in 1 s (FEV1, % predicted) of 49. At the post-intervention, the total sample is comprised 298 subjects, which represents 79,46% of retention. The selected studies were published between 1996 [21] and 2015 [22]. There were three studies from Turkey [20,22,23], and one from each of the following: USA [21], Australia [26], UK [25], Canada [27] and Spain [24]. All included studies did not register the intervention. Four studies reported the use of RE [21,22,23,26], three studies used AE [20,23] and two CE [25,27]. No study using stretching exercise met our inclusion criteria. The interventions had a duration between 6 [23,25] and 14 weeks [20]. The most frequently reported exercise frequency was three times a week [22,23,25,26], ranging between three and six days per week [24]. All interventions were performed at home, except for one study [24]. Dyspnoea was assessed in six studies using the modified Borg scale (MBS) [22], Chronic Respiratory Questionnaire-dyspnoea domain (CRQ-D) [24,26,27,32], and modified British Medical Research Council (mMRC) [23]. Fatigue was measured in seven studies using the Modified Borg scale (MBS) [20,22], Fatigue Impact Scale (FIS) [23], Fatigue Severity Scale (FSS) [23], Chronic Respiratory Questionnaire-Fatigue Domain (CRQ-F) [24,25,26,27] and Perception Fatigue Scale (BFS) [26].

### 3.3. Quality of Studies and Risk of Bias (Figure 2 and Figure 3)

Seven studies [20,24,25,27] presented a low risk due to random sequence generation and one had unclear risk of bias [23] regarding the mentioned item. Seven studies had a low risk of allocation concealment [20,21,22,23,25,26,27], and one had an unclear risk [24]. For the blinding of participants and personnel, two studies had low risk [20,21] and six studies were characterised as high risk [22,23,24,25,27]. For blinding of the outcome assessment, four studies were considered low risk [22,26,27,32], three as unclear risk [20,21,23], and one study as high risk [25]. For incomplete outcome data, seven studies had a low risk of bias [20,21,23,24,25,26,27] and one had a high risk of bias [24]. Finally, all studies were categorised as low risk for selective reporting and other bias [20,21,22,23,25,26,27].

**Figure 2:**
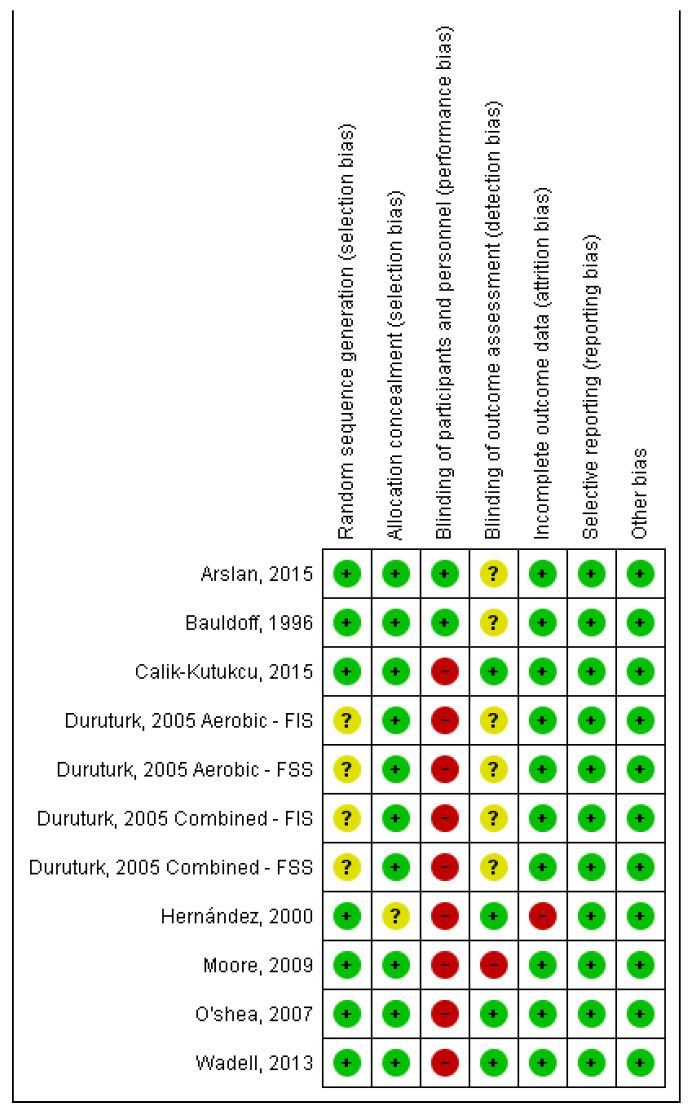
Risk of bias summary: author’s judgements on risk of bias for each included study [20,21,22,23,24,25,26,27]. Note: FIS, Fatigue Impact Scale; FSS, Fatigue Severity Scale.

**Figure 3:**
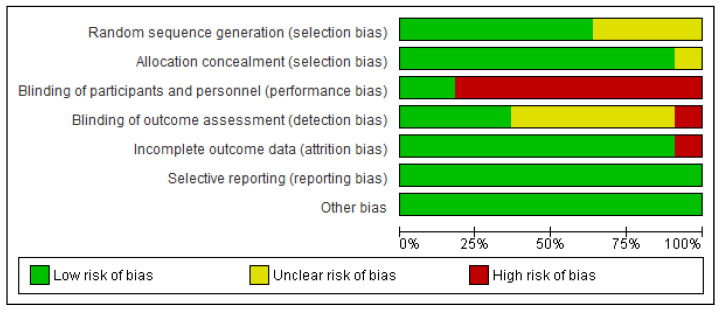
Risk of bias graph: author’s judgements on each risk of bias item presented as percentages across all included studies.

### 3.4. Meta-Analysis

**Figure 4:**
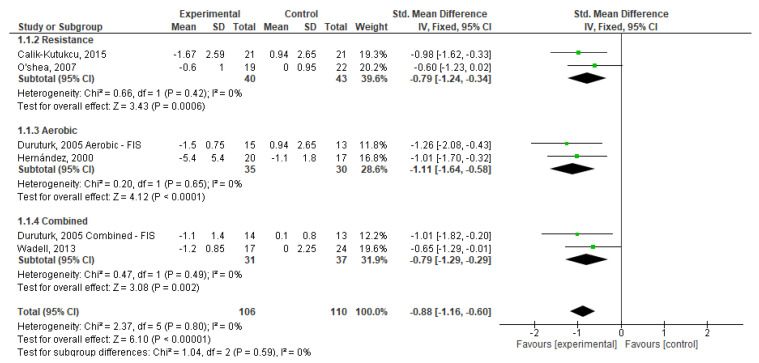
Forest plots showing the effects of exercise on dyspnoea outcome [22,23,24,26,27]. Note: SD, Standard Deviation; FIS, Fatigue Impact Scale; CI: Confidence Interval; IV, Inverse Variance.

From Figure 4, we can observe that exercise, in general, had a significant effect on dyspnoea (SMD = −0.88; 95% CI, −1.16 to −0.60; *p* < 0.001). We also found that RE (SMD = −0.79; 95% CI, −1.24 to −0.34; *p* < 0.001) and CE (SMD = −0.79; 95% CI, −1.29 to −0.29; *p* < 0.001) had a medium and significant effect and that AE had a large and significant effect (SMD = −1.11; 95% CI, −1.64 to −0.58; *p* < 0.001) on dyspnoea. Weights are from fixed effects analysis once heterogeneity was not observed (*I*^2^ < 50%, *p* > 0.05).

**Figure 5:**
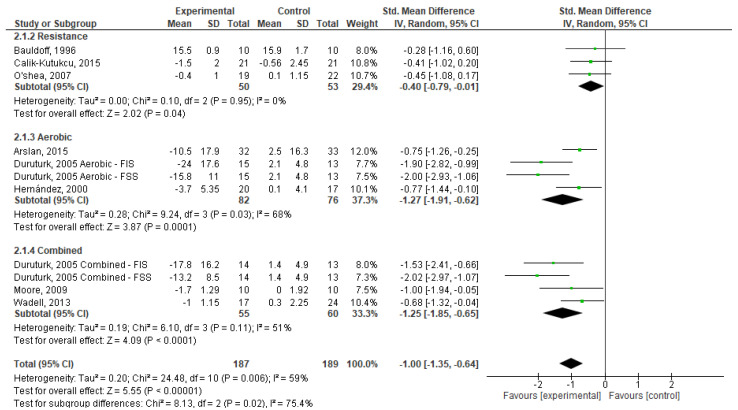
Forest plots showing the effects of exercise on fatigue outcome [20,21,22,23,24,25,26,27]. Note: SD, Standard Deviation; FIS, Fatigue Impact Scale; FSS, Fatigue Severity Scale; CI: Confidence Interval; IV, Inverse Variance.

Regarding fatigue, as seen in Figure 5, one can state that exercise, in general, had a significant effect (SMD = −1.00; 95% CI, −1.35 to −0.64; *p* < 0.001). Based on the type of exercise, RE had a small and significant effect on fatigue (SMD = −0.40; 95% CI, −0.87 to −0.01; *p* > 0.001); while AE (SMD = −1.27; 95% CI, −1.91 to −0.62; *p* < 0.001) and CE (SMD = −1.25; 95% CI, −1.85 a −0.65; *p* < 0.001) had significant effects on this symptom. Weights are from random effects analysis once heterogeneity was observed (*I*^2^ < 50%, *p* > 0.05) in AE and CE analysis.

### 3.5. Certainty of Evidence

We used the GRADE framework to evaluate the certainty of the evidence of this study. Due to methodological limitations of the included studies, the certainty of evidence in the meta-analysis of RE on dyspnoea and fatigue, AE on dyspnoea, and CE on dyspnoea and fatigue was rated as low, once we downgraded one level of certainty of the evidence, in terms of “risk of bias”, “indirectness” and “imprecision”. The evidence from the meta-analysis of AE on fatigue and CE on fatigue was rated as very low. At this point, in terms of “study design”, we identified some limitations, and downgraded one level of certainty of the evidence, in terms of “risk of bias”, “inconsistency”, “indirectness” and “imprecision”.

## 4. Discussion

Through a systematic search for RCTs, we aimed to evaluate the effect of exercise on dyspnoea and fatigue in patients with COPD. We found that eight trials involving 375 COPD patients met our inclusion criteria. The main findings of this systematic review with meta-analysis were that pulmonary rehabilitation based on exercise interventions significantly improved dyspnoea and fatigue in comparison to people that were included in the control groups benefiting from overall standard care. The findings suggest a lack of comprehensive studies comparing exercise interventions and CG. These analyses revealed moderate to substantial levels of heterogeneity and a wide range of predictive interval. There were also low to moderate heterogeneity between the studies which may indicate that there are benefits of exercise for COPD patients compared to the control groups [31,32]. Furthermore, no intervention shows a prediction interval crossing the zero line.

Some studies have already stated that exercise could be a way to promote dyspnoea and fatigue management in subjects with COPD [15,33,34]. Following our study, we could corroborate this claim, and add that the AE, RE and CE differ from each other in their impact on dyspnoea and fatigue.

Regarding RE, a significant and favourable effect was observed in dyspnoea and fatigue. Nonetheless, all RE-based interventions [21,22,26] were conducted using appendicular muscles (i.e., upper and lower limb). O’Shea et al. [23] argued that appendicular muscle RE in people with COPD can increase upper and lower limb strength. These authors also concluded that findings were inconclusive for other outcome measures, such as aerobic capacity, and psychological and respiratory function. However, according to renowned exercise testing and prescription guidelines [13,14], RE should be encouraged for individuals with COPD, and exercise prescription for this population should follow the same FITT (i.e., frequency; intensity; type; and time) principle used for healthy adults and/or older adults. This recommendation calls for a focus on the major muscles, which we did not observe in the studies included in the present review regarding the effect of RE on dyspnoea and fatigue. Despite the fact that RE interventions were directed for lower and upper limbs, the results obtained showed that RE for upper and lower limbs can also improve dyspnoea and fatigue in this population.

Therefore, RE interventions targeting the upper and lower limbs, in addition to promoting strength in these muscle groups [26], also contribute positively to improving quality of life through better management of dyspnoea symptoms and fatigue. It should also be noted that this type of exercise was the one that “produced” the smallest effect in both COPD symptoms, of all the types of exercise included in this review.

Regarding AE interventions, a significant effect bettering fatigue and dyspnoea was observed in all subjects with COPD. All studies included in the AE analysis [20,23,24] showed improved dyspnoea and fatigue symptoms. There are several challenges for exercise prescription and physical activity (PA) participation in this population, but a substantial evidence demonstrates important health benefits of aerobic exercise, including decreases in dynamic hyperinflation and exertional dyspnoea, improved exercise tolerance, and enhanced quality of life with fewer disease exacerbations and reported sick days [5]. Worldwide renowned exercise testing and prescription guidelines [13] suggest that AE should be performed by walking or cycling 3–5 days a week with vigorous intensity (60–80% of peak work rates) and light intensity (30–40% of peak work rates). Due to better results on physiologic capacity (e.g., minute ventilation), vigorous intensity should be encouraged if tolerated. The light intensity of AE is too an option since it also improves symptoms and quality of life. Considering that the exercise prescription of AE interventions included in our analysis followed renowned exercise testing and prescription recommendations [13], we may state that our results corroborate such recommendations for controlling dyspnoea and fatigue in this population.

A significant and favourable (positive) effect on the studied COPD symptoms were also found in two include studies using CE interventions. In Wadell’s [27] study, the intervention was conducted with the combination of AE and RE. These findings support the assertion that an intervention based on the combination of both types of exercise on COPD patients is an effective method to promote desirable clinical benefits. Further benefits have been reported, including walking capacity, body composition, health state perception, and quality of life in subjects with COPD [35,36]. In Durututk’s [23] study, CE intervention was conducted with rhythmical callisthenic exercises that included strengthening and stretching of the lower and upper extremities. Typically, due to disease characteristics, this population only performed respiratory muscle stretching after hyperinflation of the lungs resulted in remodelling of the inspiratory muscles, particularly the diaphragm, which may become depressed and may have reductions in movement [37,38]. This activity increases the capacity for chest wall expansion, suggesting an improvement in ventilation in patients with COPD [39].

However, stretching exercises should focus on major muscle-tendon units and not only on respiratory muscle [13,14]. Furthermore, stretching exercises may acutely reduce power and strength, so it is recommended that flexibility exercises must be performed after any exercise and sports [13]. It could explain why our inclusion criteria did not find a single stretching-based intervention. Stretching-only interventions are uncommon in this population, except for respiratory muscle stretching interventions. This is reinforced by the fact that in 2013, Gloeckl, Marinov, and Pitta [6] suggested various types of exercise (e.g., resistance and strength) without considering stretching exercise in their recommendations to a rehabilitation programme in COPD subjects.

We discovered that our search yielded fewer RCTs than other meta-analysed systematic reviews [15,16]. However, our study was the first to investigate interventions that only used exercise, excluding interventions that used other common pulmonary rehabilitation strategies (e.g., breathing education and massage therapy); this could explain the differences in the number of included studies.

We also observed that most interventions were home-based [20,21,22,24,25]. This factor might be relevant since Gold [1] suggests that this type of intervention should be prescribed in some situations. In the recent COVID-19 pandemic, for example, many pulmonary rehabilitations were suspended to reduce the risk of spreading SARS-CoV-2. In this situation, centre-based rehabilitation is not appropriate, and patients should be encouraged to keep active at home and be supported by home-based rehabilitation programmes [1]. Our findings highlight the use of home-based interventions after confirming that most of the interventions included in our study [20,21,22,24,25] produced a positive effect on COPD symptomatology.

Therefore, we suggest that healthcare and exercise professionals could create conditions where this population could remain active, and get involved in programmes of AE, RE and CE, where the prescription of exercise for healthy/older adults has been established in line with ACSM [13].

This review and meta-analysis have certain strengths and limitations. A sound methodology was followed, and the research question was specific enough to draw important outcomes with relevance for clinical practice and for the benefit of the target population. We acknowledge some limitations that should be pointed out. In accordance with the GRADE framework, we rated the certainty of the evidence as low to very low due to methodological limitations in the included studies. Additionally, although our meta-analysis obtained very satisfactory results, it was only conducted with a small number of studies for each exercise intervention. Therefore, more accurate studies need to be conducted in the future with larger samples to accurately identify and understand the effects of intervention exercises on dyspnoea and fatigue in COPD subjects and to reduce the bias associated with studies for this population. This will subsequently lead to a fitting and extensive use of exercise as a means of promoting quality of life in persons with COPD.

## 5. Conclusions

The aim of this study was to summarise, through a systematic review and meta-analysis, the available evidence on the effects of exercise interventions on the main symptoms of COPD, dyspnoea and fatigue. The results indicate that exercise intervention can effectively improve dyspnoea and fatigue in persons with COPD. It was also observed that the three types of exercise (AE, RE, and CE) had a significant positive effect on dyspnoea and fatigue. Results indicate that RE had a medium effect on dyspnoea and a small effect on fatigue; AE had a large effect on dyspnoea and an even larger effect on fatigue; CE had a medium effect on dyspnoea and a very large effect on fatigue.

## Figures and Tables

**Table 1:** Search strategy by PICOS.

Population	Subjects aged 18 years old or older, diagnosed with COPD at moderate and severe stage of severity, in a stable phase of the disease (i.e., four weeks without hospital admissions or exacerbations, nor changes in medication), according to Gold [1].
Intervention	Randomised control trials (RCTs) with interventions based on one of the following types of exercise: aerobic (AE), resistance (RE), stretching (ST) or combined (CE).
Comparison	All studies including a comparison of subjects that performed at least one type of exercise (i.e., AE, RE, ST, and CE), with others who maintained their daily activities with standard care for COPD.
Outcomes	Studies assessing the effects of exercise on dyspnoea or fatigue.
Type of Study	RCTs comparing AE, RE, ST or CE with a control group receiving no treatment or standard care were included.

**Table 2:** Characteristics of the included studies.

Author/Year	Participants	Age (Years) (M ± SD)	Intervention	Outcomes	Conclusions
Arslan and Oztunc 2015 [20]	AE: 32 subjects CG: 33 subjects	AE = 56.9 ± 6.6 years CG *	Intervention: AE. Patients walked three days a week with low to moderate intensity. Comparison: treatment-as-usual (CG). Duration: 45 min. three times a week for 14 weeks.	Fatigue-Modified Borg Scale	Compared to CG, walking exercise programme applied to patients with COPD affected the fatigue symptom positively.
Bauldoff et al., 1996 [21]	RE: 10 subjects CG: 10 subjects	RE = 61 ± 14 years CG = 63 ± 13 years	Intervention: RE. Patients performed three sets of six repetitions of arms and shoulders exercises. The training level (weight used, sets, and repetitions) for the muscle groups began at a low stage and progressed, according to subject tolerance. Comparison: treatment-as-usual (CG). Duration: five times a week for eight weeks.	Fatigue-BFS	A home-based, upper-arm exercise programme can reduce perceptions of fatigue for patients with COPD.
Calik-Kutukcu et al., 2015 [22]	CE: 28 subjects SE: 28 subjects	RE = 58.38 ± 9.32 years CG = 59.71 ± 9.3 years	Intervention: RE. Exercise was conducted with free weights at 40–50% 1 RM, three sets/session, three times/week, with 8–12 reps loading for a total 23 supervised sessions over an eight-week period. Comparation: treatment-as-usual (CG). Duration: three times a week for eight weeks.	Fatigue-Modified Borg Scale Dyspnoea-BDI	Muscle strength exercise decreases dyspnoea and arm fatigue perception during supported arm exercises, and dyspnoea perception during daily living activities.
Duruturk et al., 2005 [23]	CE: 16 subjects AE: 16 subjects CG: 15 subjects	CE = 61.2 ± 5.1 years AE = 61.2 ± 5.0 years CG = 63.8 ± 5.7 years	Intervention: CE. The subjects performed 16 different rhythmical, and calisthenic exercises that included strengthening and stretching of the lower and upper extremity muscles. The intensity of the calisthenics was adjusted using the Modified Borg Scale to maintain a perceived difficulty level of between 4 and 7. The intensity of the aerobic exercise intensity was also adjusted based on the subjects’ Modified Borg Scale rated dyspnoea or leg fatigue. The intensity was increased if the Borg rate was <4, and decreased if it was >7. Comparation: treatment-as-usual (CG). Duration: three times a week for six weeks. Intervention: AE. The subjects performed cycle ergometer exercise (Dunlop Sport 1696 Cycle Ergometer, Japan) consisting of 20–30 min of continuous cycling at 50–70% of the VO2max obtained from the cycle ergometer exercise testing. Comparation: treatment-as-usual (CG). Duration: three times a week for six weeks.	Dyspnoea-MMRCDS Fatigue-FIS; FSS	The dyspnoea and fatigue changed significantly in exercise groups. There were no significant improvements in control group.
Hernández et al., 2000 [24]	AE: 30 subjects CG: 30 subjects	AE = 64.3 ± 8.3 years CG = 63.1 ± 6.9 years	Intervention: AE. The intensity of walking was set at 70% of the maximum speed attained on the shuttle walking test (SWT). Comparison: treatment-as-usual (CG). Duration: AE: six times per week (60 min) for 12 weeks.	Dyspnoea-BDI	AE exercise achieved improvement in dyspnoea and quality of life in COPD patients.
Moore et al., 2009 [25]	CE: 10 subjects CG: 10 subjects	CE = 70 ± 13 years CG = 70.5 ± * years	Intervention: CE. Warm-up, high-intensity interval exercises (upper and lower limb strengthening and aerobic exercise) and a cool-down including stretches. Free weights and body weight were used for strengthening. The subjects were educated to achieve the status of intensity “somewhat severely out of breath” and “somewhat severely tired”, corresponding to a Borg score of four. Comparison: treatment-as-usual (CG). Duration: four times a week (30 min) for six weeks.	Dyspnoea-CRQ-D Fatigue-CRQ-F	The experimental group had improved on dyspnoea and fatigue.
O’Shea et al., 2007 [26]	RE: 27 subjects CG: 27 subjects	RE = 66.9 ± 7.0 years CG = 68.4 ± 9.9 years	Intervention: RE. Progressive resistance exercise of three sets of eight to 12 repetition maximum progressed against elasticised bands. Comparison: treatment-as-usual (CG). Duration: three times a week for 12 weeks.	Dyspnoea-CRQ-D Fatigue-CRQ-F	The experimental group had improved on dyspnoea and fatigue.
Wadell et al., 2013 [27]	CE: 20 subjects CG: 20 subjects	CE = 68 ± 6.0 years CG = 66 ± 7.0 years	Intervention: CE. Sessions included graduated exercise for upper and lower limbs-walking on treadmill and in corridor, cycle ergometer, arm ergometer, strength/resistance exercises for upper and lower limbs. Subjects worked at their highest attainable work rate for the longest tolerable duration by targeting at least a “moderate” intensity of breathing discomfort on the modified 10-point Borg scale. Comparison: treatment-as-usual (CG). Duration: programme consisted of three supervised 2.5 h sessions per week over an 8-week period.	Dyspnoea-CRQ-D Fatigue-CRQ-F	The experimental group had improved on dyspnoea and fatigue.

Note: M, mean; SD, standard deviation; * not available; AE, aerobic exercise intervention; RE, resistance exercise intervention; CE, combined exercise intervention; CG, control group; 1 Rm, of one repetition maximum; BFS, Perception Fatigue Scale; BDI, Mahler’s Basal Dyspnoea Index; MMRCDS, Modified Medical Research Council Dyspnoea Scale; FIS, Fatigue Impact Scale; FSS, Fatigue Severity Scale; CRQ-D, Chronic Respiratory Questionnaire-Dyspnoea Domain; CEQ-F, Chronic Respiratory Questionnaire-Fatigue Domain.

## Data Availability

Data are available under request to the first author.
